# Equivalent sentiment measures for cross-language analysis of corporate communications

**DOI:** 10.1016/j.mex.2024.102745

**Published:** 2024-05-10

**Authors:** Karol Marek Klimczak, Jan Makary Fryczak, Dominika Hadro, Justyna Fijałkowska

**Affiliations:** a Institute of Management, Faculty of Organization and Management, Lodz University of Technology, Wólczańska 221, 93-005, Łódź, Poland; b Faculty of Economics and Finance, Wroclaw University of Economics and Business, Komandorska 118/120, 53-345, Wrocław, Poland; c University of Social Sciences, Sienkiewicza 9, 90-113, Łódź, Poland

**Keywords:** Wordnet-based translation of financial dictionaries for textual analysis, Business communication, Financial reporting, Textual analysis, Text mining, Python

## Abstract

This paper presents a technique for sentiment measurement in many languages. The method allows researchers to efficiently analyze corporate documents, management reports, and financial statements using python. When the texts are written in many languages, the method extracts equivalent cross-linguistic sentiment features that can be used for statistical analysis or machine learning. We use Open Multilingual WordNet, a large lexicon organizing words into semantic groups, as the knowledge base about word equivalence in more than 200 languages. We experiment with a parallel English-French corpus and find that our senitment measures across the two languages are comparable. The method produces a consistent classification of positive and negative texts in two languages, and sentiment measure values correlate. The paper provides a detailed account of the method and python code, So that it can be applied to other languages, text mining, quantitative communication studies, and management research.

• Method to create equivalent sentiment measures in multiple languages
• Based on established lexicons and WordNet
• Validated for English and French

## Graphical abstract

**Figure:**
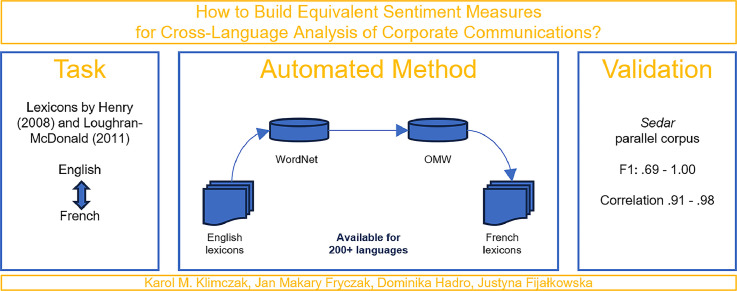

Specifications table

**Table:** 

Subject area:	Economics and Finance
More specific subject area:	Accounting
Name of your method:	Wordnet-based translation of financial dictionaries for textual analysis
Name and reference of original method:	N/A
Resource availability:	https://github.com/kmk4842/opus2021 https://github.com/autorite/sedar-bitext

## Measuring sentiment in financial texts

Researchers and businesspeople study corporate communications and reports to learn about the management, strategy, company financial results, and build expectations about future business performance [1,2]. With advances in natural language processing and artificial intelligence, it has become possible to study large volumes of company documents using computer-aided methods, with significant gains in data processing efficiency. International studies remain surprisingly rare, however, because existing methods are limited to one language [3,4]. In fact, English is the language with highly developed textual analysis tools, such as multidisciplinary sentiment lexicons and large corpora in digital formats. The purpose of this paper is to introduce a method for building new resources in languages beyond English. We use the French language as an example, but our technique can easily be adapted to other languages following the steps described in this paper. We provide code in python at https://github.com/kmk4842/opus2021.

The purpose of this paper is to describe a method for constructing sentiment measures in two or more languages that would produce comparable values, thus facilitating international research. The method recombines available textual analysis resources in a novel way. We show how to automatically translate English sentiment lexicons (i.e. word-lists), well established in the literature, to French using WordNet as the knowledge base about word equivalence [5]. WordNet is a lexical database organized in sets of synonyms, i.e. synsets, which carry information about shared meanings of particular words [6]. National Wordnets (French WOLF, GermaNet, Polish plWordNet, Spanish/Italian MCR and more) are mapped between each other using an interlingual index (ILI) that references synsets from one language to another [7]. Our mapping procedure yields lists of words in two languages that can be used to generate comparable sentiment measures. We evaluate the equivalence between measures in English and French using a parallel English-French SEDAR corpus made available by L'Autorité des Marchés Financiers in Québec, where companies are required to prepare annual statements in two languages [8]. The results show a high degree of consistency between two languages in both classification and feature values.

Our method for mapping lexicons between languages provides a transparent setting for translating word-lists to new languages, manipulating and evaluating versions of new lexicons. It offers several improvements over existing approaches. First, we integrate translation into the research method by using Open Multilingual WordNet, rather than relying on translation of original texts by human or machine. The process is more stable and verfiable. Second, the procedure is transparent, making it possible to extend it to other languages with little adaptation work required. Third, we apply our method to extend not only the Loughran and McDonald [9] lexicon but also Henry [10], which has been shown to be more efficient. In addition, readers may apply this method to other word-lists they find useful in research. Finally, this is the first paper to present sentiment measurement results for the French language using a unique parallel corpus of financial statements published in both English and French by Canadian companies.

## Related work

The measurement of sentiment has a long tradition, hence we set well-established measures as the starting point for the presentation of our method. The first text analysis software used extensively in finance and accounting sentiment research were DICTION [11,12], and General Inquirer Harvard IV dictionary [13]. In 2006, Elaine Henry published an article on investors' reactions to earnings press releases including a dictionary, which she later developed in Henry [10]. Her dictionary contains 211 words, including 118 positive and 93 negative words. She measured sentiment using equally weighted word frequencies.

In 2011, Loughran and McDonald published sentiment analysis results of U.S. annual 10-K reports published between 1994 and 2008. They compiled a list of words appearing in these reports along with their frequencies. Loughran and McDonald [9] prepared lists of positive and negative words, and later expanded their research to include uncertainty, litigious language, strong and weak modal words, and, more recently, constraining language. In this paper, we focus on the positive and negative lists only. The first wordlist contained 2337 negative words, about half of which (1121 words) corresponded to Harvard General Inquirer lists. Over the years, the lexicon posted on the authors' website increased to 2354 negative words. The number of positive words is much lower, only 353, reflecting the assumption of its creators that negative words have a much more pervasive effect and are crucial in evaluating the firm value.

Henry and Leone [14] and Young et al. [15] evaluated various tools to compute sentiment in corporate communication. They confirm that domain-specific lexicons [[9], [10]] outperform general ones (DICTION and Harvard General Inquirer), because they mitigate polysemy. Young et al. [15] claim that Henry's [10] wordlist gives more reliable results than Loughran and McDonald [9], while Henry and Leone [14] do not find significant differences. In this paper, we study both lists.

With rare exceptions, extant research is confined to the English language. Two articles use domain-specific sentiment wordlists to evaluate non-English financial reports, although they differ substantially from ours in the method. First, González et al. [16] measure the sentiment of letters to shareholders published by Latin American companies in Spanish and Portuguese. Their wordlists for Spanish and Portuguese from Loughran and McDonald [9] lexicon using an automatic language translator were polished manually to control for duplications and ambiguous words. Second, Bannier et al. [17] adapt the same lexicons for German using word-by-word manual translation to control differences in inflectional morphology, lexical morphology, and compound wording. To evaluate their lexicon, they compare sentiment measures of financial reports in German with convenience translations provided by the same companies, obtaining a correlation coefficient above 0.7. Their results suggest that the construction of equivalent sentiment measures across languages is indeed possible. We follow their steps by using a parallel corpus of English-French reports published by Canadian companies. Third, it is possible to build a sentiment lexicon from the ground up, for example, by finding which words appear more often when companies record positive vs. negative returns in the stock exchange [18]. In that case, however, cross-language studies are not facilitated.

What our method contributes to the state-of-the-art is a bridge between non-English texts and machine learning based on a transparent process. As mentioned above, English texts have been used in studies of global markets with machine-learning methods [19], where machine-translation is the most common approach to the study of non-English texts [20]. Text-based features sourced from machine-translation to English do increase forecast accuracy in practice [21]. What that approach lacks is transparency in the treatment of differences in how sentiment is expressed in other languages, and these differences can be striking in langauages as far from English as Chinese. For that reason, management researchers avoid the use of machine-learning. Limited replicability is another concern. The recent boom in generative artificial intelligence may open new avenues for textual feature extraction, but these are yet to be applied in social sciences [22]. In sum, our method enables researchers to expand their studies to an international level by allowing them to measure sentiment across languages.

## Steps of the method

### Mapping financial lexicons to wordnet

**Fig. 1:**
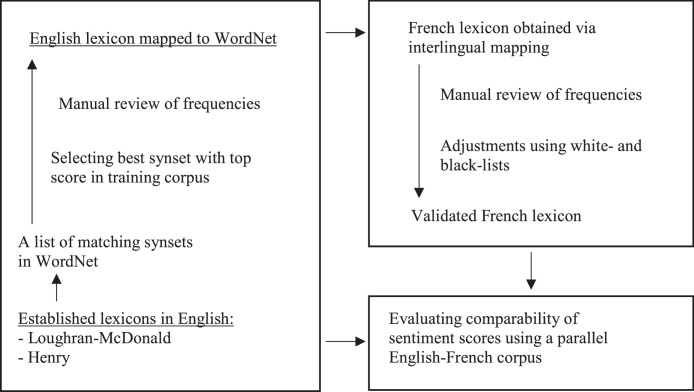
Mapping sentiment lexicons from English to French.

We begin by creating semantic links between the original word-lists [[9], [10]] and the Princeton WordNet (PWN). This process needs to be performed only once, unless one looks to tweak the mapping. An outline of all the steps is presented in Fig. 1. The python code includes the entire process transparently. The first step is the identification of PWN synsets (i.e. sets of synonyms) that include each word using a process called lemmatization that yields a lemma (a standardized form of a word). Thus, each word (or, more formally, each lemma) is assigned a list of synsets (if any matching synset exists). Note that lemmatization based on WordNet is an improvement over stemming that would bring related words to the common stem or core regardless of their meaning.

The second step involves selecting the best synsets from the set of matching synsets obtained during lemmatization. How can an algorithm determine which meaning is the most appropriate? The logic of PWN provides an answer: each synset contains synonyms beyond the one word from the original list. Therefore, it is logical to select the synset containing words that appear with the greatest frequency in a training corpus. A high frequency suggests that words linked to this meaning are the most pertinent in the context of interest. The lemma “gain”, for example, appears in four noun synsets, but only one of them includes the lemma “profit”, which is highly relevant in business contexts. When we examine the results from the training corpus, lemmas from that single synset appear with a frequency six times larger than lemmas from the remaining synsets.

Once the selection of synsets is complete, we obtain a WordNet version of the original lexicon: a list of synsets representing concepts relevant to the sentiment analysis task. The final step is output generation for the task at hand. A simple list of lemmas is sufficient for most uses since texts in the test corpus were lemmatized during pre-processing. We obtain lemmas for each synset, adding them to the sentiment lexicon and removing duplicates. Next, the lemmas need to be verified manually. In some cases, a synset might include a common word that is used very often and takes various meanings. For example, the words “sue” and “litigate” that appear on the negative-sentiment list indicate the synset “action.v.01” as relevant. That synset, however, includes the word “action”, obviously, which is used in a variety of meanings, not uniquely negative ones. We include such lemmas in a black-list to remove them from further analysis. In the following section, we report relevant diagnostic tests to evaluate the output.

It is important to note the reasons for dropping terms from the original lexicon. This occurs when there is no matching synset, or lemmas from matching synsets do not appear in the training corpus. First, the lack of a matching synset suggests that the original word may be too subject-specific and narrow, thus not appearing in WordNet. In fact, the WordNet community decided to distinguish the lexicon from an encyclopedia by, for example, excluding terms that appear in Wikipedia [23]. This occurs in several cases, such as “bailout”, “illiquidity” or “writedown”. Another reason for missing synsets may be word-creation. WordNet avoids creating additional synsets for words resulting from a straightforward derivation without changing the meaning, such as negation via prefix “un” or adverbs derived from adjectives via suffix “-ly”. These lemmas can be added manually to the final list. Second, if a word is missing from the training corpus, it is not relevant for the task at hand. The word can safely be dropped then, but care must be taken to re-evaluate such cases when switching to another corpus where they might appear again.

### Translating lexicons

We use Open Multilingual WordNet (OMW) to translate the original lexicons in English to French – a step that needs to be repeated for each new language. The method is outlined in Fig. 1. First, we find French synsets matching each of the English synsets identified in the previous step. OMW offers simple and well documented access to interlingual mapping by referencing a synset by its name and adding the ISO-639 code for the foreign language, e.g. calling synset(“sucess.n.03”, lang = “fra”) returns a French language synset that corresponds to “success.n.03” PWN synset. The direct reliance on synsets identified in the previous step implies that the list of synsets must be verified and of high quality. Second, we generate a list of lemmas from each French synset so that we may obtain their frequencies.

The following issues arise at this stage. First, interlingual mapping in OMW is highly simplified. Individual, national WordNets, grouped in the Global WordNet Association (http://globalWordNet.org), may offer richer links to English but at the cost of more complicated access. Recently, the “wn” python package introduced a new integrated interface to many WordNets, which offers several advantages over the “nltk” implementation. It allows each WordNet to have its own structure rather than enforcing the PWN structure on all WordNets [24]. Moreover, WordNets in “wn” are transparently versioned, while version information is obscured in the standard “nltk” package. The user can add any WordNet in WN-LMF format, making “wn” more customizable. Nevertheless, we limit the presentation in this paper to OMW for the sake of simplicity.

Second, the structure of PWN itself imposes certain limits on translation. For example, PWN includes relatively few adverb synsets because adverbs in English are created from adjectives using a simple rule: adding the suffix “-ly” (there are exceptions, e.g. “good” and “well”). The meaning is not modified; hence no new word-sense needs to be included in WordNet. Fortunately, the French WordNet included in OMW (WOLF) takes a similar approach, resulting in a good match during translation. Should a work-around be necessary, French adverbs are also derived from adjectives with the suffix “-ement”, as is the case with most European languages. When considering other languages, it may be better to rely on the national WordNet for adverb synsets. Notably, plWordNet includes more than twice as many adverb synsets in Polish as PWN in English [25]. In our case, since we use OMW for both language versions, adverbs are lemmatized to the underlying adjective form. No work-around is necessary.

Third, when synsets in two languages do match conceptually, there may still be differences in conditional frequencies. An analysis of lemmas included in matching synsets shows that the creators of the French WordNet solved this problem by including more lemmas, broadening original PWN synsets. The synset “large.a.01”, for example, includes two lemmas, “large” and “big” in PWN, but the French synset includes four lemmas: “grand”, “gros”, “large”, and “nombreux”, thus broadening the meaning to encompass the concept of size determined by width and number. Moreover, some lemmas may be used much more often in one language, potentially leading to other synsets being more relevant than the ones obtained via the link to PWN [26]. The preferred solution depends on the part of speech, as WordNet provides much more information on verbs and nouns than on adjectives and adverbs. Verb and noun synsets are organized in a tree-like structure, making it possible to find a better synset, appearing with the desired frequency in the training corpus among sister synsets that share the same hypernym. Adjectives, in contrast, are organized around antonym pairs via similarity relations, which are rather broad, making it difficult to identify relevant synsets. When such cases occur, manual corrections are necessary. For simplicity, we limit manipulations to creating a black-list of lemmas that need to be removed, and a white-list of lemmas that need to be included in French lexicons as described below.

We adopted the following manual revision protocol for each French lexicon. Initially, we verified synset mapping by comparing the total frequencies for the English and French versions of the synset, as well as the composition of each pair of synsets. This step revealed that several French synsets are empty. Apparently, these particular meanings do not appear to be distinguished in French or there is no single word in French for these concepts. The synset “charitable.s.03” from the positive lexicon, for example, contains seven lemmas in PWN, but none in the French WordNet, though there do exist French lemmas for the other two “charitable.s.” synsets. We solved this problem by preparing a white-list of lemmas that must be included even if a synset is missing. Another issue is the presence of general words in some French synsets, such as “avoir” (to have) in “interview.v.01”, and a handful of apparent errors, such as “puits” (a well) in the adverb synset “well.r.11”. We solved such problems by extending the black-list of lemmas that must be removed. For final corrections, we verified the lemmas again, looking at each of them in turn and considering their frequencies in the training corpus while revising the black- and white-lists. In sum, manual corrections are necessary, but the initial algorithmic processing expedites the entire process, helps assure transparency, and replicability.

## Method validation

This section describes a test of the method using a parallel English-French SEDAR corpus, which contains pairs of sentences drawn from financial statements filed by companies in Québec, where filings need to be prepared in two languages. The corpus can be obtained from L'Autorité des Marchés Financiers [8]. The entire corpus contains 8.6 million sentence pairs sourced from filings that each listed company in Québec makes in two language versions. The authors of SEDAR took care to align sentences, provide quality measures for each pair, and split the corpus into the train, validate, and test parts. For this research, we draw two random samples of 70,000 sentence pairs from the training sub-corpus, which counts a total of 7 million sentence pairs.

We evaluate the mapping from the original lexicon to PWN by comparing results based on the seed lexicon to those based on the WordNet lexicon. This involves measuring the frequency of positive and negative words in each sentence (typical counts are one, two, or three occurrences in a sentence) and then calculating the net-positivity index as the difference between positive and negative frequencies divided by their sum [1]. We use TF-IDF frequencies instead of counts in line with the original research. Then, we classify sentences containing as positive or negative, depending on the presence of lemmas from either the positive or negative lexicon. In addition, we classify sentences as net-positive or net-negative, depending on the sign of the net-positivity index. Finally, we calculate standard classification statistics for discrete data (classifications), and descriptive and correlation statistics for interval data (net-positivity index values). Having completed this initial evaluation, we proceed to compare the results between pairs of English and French sentences.

Validation involves Henry [10] and Loughran and McDonald [9] lexicons, which we refer to as Henry and LM respectively. We map each word in the seed lexicon to PWN, associating it with a synset. Then, we generate English and French lexicons based on the synsets. Manual corrections comprise a black- and a white-list of lemmas, as described in the previous section.

In the case of LM, the total count of lemmas decreases from 320 to 198 in the positive lexicon because the remaining lemmas do not appear in the training corpus with the minimum required frequency, which we set to 10. Two words from the original lexicon lack synsets: “proactively” and “exclusivity”. Only the latter appears in the corpus at all, and even then, only fifteen times, thus the two words appear irrelevant and can safely be dropped. Mapping the negative list is more complicated because it includes diverse words and their derivations, which do not appear in the training corpus. As a result, the WordNet lexicon contains 540 lemmas compared to 2001 in the original lexicon. Over a thousand original lemmas are totally missing from the corpus. In addition, 117 lemmas do not match any synset, because they are either unusual (e.g. “irreconcilably” or “unreasonableness”) or represent business-related concepts that are excluded from WordNet by design as context-specific, such as “write-off” or “illiquid”. These could be translated manually to French, but we decided such interventions are out of scope for the present research.

In comparison, Henry's [10] lists are more cohesive, resulting in straightforward mapping to PWN. The number of lemmas decreases in both the positive (74 from 93) and negative lexicon (50 from 71). No original lemmas miss synsets to match.

We proceed with the evaluation of the equivalence between the original and WordNet lexicons in English. First, we visualize the results by comparing the top thirty words by frequency in Table 1, which shows a high degree of equivalence. Nevertheless, WordNet lexicons include some additional lemmas, because they share synsets with the original ones, i.e. they are synonyms. For example, the positive list of Henry does not include the lemma “gain”, but it does include “increase”, which happens to be the most common lemma. The mapping algorithm identified synset “addition.n.03” as the most relevant, thus including in the lexicon the following synonyms: “increase”, “gain” and “addition”, the last of which was black-listed, however, as a general term. In this way, mapping to WordNet thus results in a slight extension of the original lexicons.

**Table 1:** The top 30 lemmas in the English lexicons.

Panel A: Loughran-McDonald Word-List												
Positive							Negative					
**WordNet-based**				**Original**			**WordNet-based**				**Original**	
high		100		gain		100	loss		100		loss	100
respect		72		benefit		71	impact		71		closing	34
gain		70		effective		59	closing		34		decline	30
purpose		51		strong		44	decline		30		claim	20
benefit		49		good		39	claim		20		defer	20
complete		47		opportunity		32	defer		20		concern	18
effective		41		improve		30	concern		18		impairment	17
strong		31		achieve		29	impairment		17		negative	15
good		27		positive		28	negative		15		terminate	14
amend		27		great		23	terminate		14		volatility	13
meet		27		able		18	volatility		13		adverse	13
lead		24		improvement		18	adverse		13		disclose	13
opportunity		22		enable		14	disclose		13		termination	13
improve		21		despite		13	restriction		13		restructuring	11
achieve		20		advance		13	termination		13		default	10
special		20		enhance		12	bear		12		adversely	9
ensure		20		attractive		11	restructuring		11		limitation	8
positive		20		success		11	uncertainty		11		restate	8
exceed		19		beneficial		11	default		10		expose	8
rise		18		successful		10	adversely		9		cease	8
profit		18		advantage		10	fall		9		delay	7
award		17		profitability		9	differ		9		weak	7
great		16		progress		9	vary		9		failure	7
plus		16		strengthen		9	limitation		8		conflict	6
well		14		strength		9	restate		8		detract	6
resolution		13		satisfy		9	consequence		8		unable	6
able		13		efficiency		9	expose		8		fail	6
improvement		12		beneficially		8	cease		8		slow	6
reach		12		stable		8	delay		7		cut	5
secure		11		pleased		7	restrict		7		challenge	5

Panel B: Henry [10] Word-Lists												
Positive							Negative					
**WordNet-based**			**Original**				**WordNet-based**			**Original**		
increase	100		increase		100		risk	100		risk		100
high	52		high		52		decrease	67		decrease		67
certain	44		certain		44		low	60		low		60
growth	42		growth		42		decline	26		decline		26
gain	36		record		29		negative	13		negative		13
record	29		strong		16		uncertainty	10		uncertainty		10
complete	24		good		14		fall	8		fall		8
large	19		opportunity		11		weak	6		weak		6
strong	16		improve		11		failure	6		failure		6
good	14		deliver		11		lower	5		lower		5
develop	13		achieve		11		fail	5		fail		5
lead	13		positive		10		challenge	5		challenge		5
opportunity	11		exceed		10		difficult	4		difficult		4
improve	11		rise		9		penalty	4		penalty		4
deliver	11		grow		9		weakness	3		weakness		3
achieve	11		improvement		6		unfavorable	3		drop		2
positive	10		expand		5		less	3		weaken		2
exceed	10		expansion		5		bankruptcy	3		uncertain		2
rise	9		success		4		modest	3		downturn		2
grow	9		successful		4		drop	2		difficulty		2
great	8		progress		3		bad	2		risky		1
well	7		strengthen		3		reduced	2		deteriorate		1
improvement	6		strength		3		weaken	2		challenging		1
reach	6		solid		3		uncertain	2		down		1
extend	5		leader		3		downturn	2		disappointing		1
expand	5		pleased		3		downward	2		unfavorable		1
expansion	5		achievement		2		difficulty	2		weakening		1
success	4		reward		2		dispute	2		threat		1
successful	4		excellent		2		risky	1		worsen		1
advantage	4		improved		2		deteriorate	1		depressed		0

*Note:* Table shows relative counts, setting the top most frequent lemma to count 100.

Second, classification statistics in Table 2 confirm that the WordNet lexicon includes some additional lemmas, thus classifying more sentences as positive or negative compared to the original word-list. Recall ratio values (0.97 to 1.00) are higher than precision ratio values (0.53 to 0.95) as a result. The results are stronger for Henry lexicons, with a precision of 0.77 for the positive lexicon and 0.95 for the negative lexicon, compared to 0.53 and 0.80 for LM, respectively. However, when we classify sentences based on the balance of positive and negative lemmas, both lexicons perform well, with the F1-score of 0.98 for LM and 1.00 for Henry.

**Table 2:** Classification statistics for WordNet lexicons by tone class.

	Precision	Recall	F1	Macro F1	Kappa
Loughran-McDonald					
Positive	.53	.99	.69	.86	.60
Negative	.80	.97	.87	.95	.84
Net-Positive	.95	1.00	.97	.97	.94
Net-Negative	1.00	.95	.97	.97	.94
Henry et al. [10]					
Positive	.77	1.00	.87	.93	.82
Negative	.94	1.00	.97	.99	.97
Net-Positive	1.00	1.00	1.00	1.00	.99
Net-Negative	1.00	.99	1.00	1.00	.99

*Note:* Table shows the comparison between original word-list and the respective WordNet lexicon based on a corpus of 69,913 sentences. Precision is the ratio of true positives to all positives, Recall is the ratio of true positives to the sum of true positives and false negatives, F1 is the harmonic mean of precision and recall, Macro F1 is the average of scores for all classes (e.g. positive and non-positive), Kappa is Cohen's kappa score.

Third, we calculate net-positivity ratios and compare their values between lexicons in Table 3. The statistics show an important level of equivalence, with mean ratio values separated by a fraction of variance, although still significant statistically given a large sample. The original LM lexicon generates a slightly negative mean ratio value because the negative list is much longer than the short one. The WordNet lexicon, which is shorter, results in a slightly positive mean ratio value. In contrast, Henry's list is more balanced, resulting in a positive mean value reflective of the generally positive tone of corporate communications described in the literature. Correlation coefficients between ratio values for the original and WordNet lexicons are high and significant, at above 0.9, although lower for LM. These results were obtained with TF-IDF frequencies, but the results for simple counts are nearly identical. We do not reproduce them here.

**Table 3:** Descriptive statistics of net positivity ratio by lexicon.

	Mean	SD	Corr.	N
Loughran-McDonald				
Original lexicon	−0.07	.93	.91	22,438
Wordnet lexicon	.03	.87		
Henry et al. [10]				
Original lexicon	.43	.83	.98	20,350
Wordnet lexicon	.45	.81		

*Note:* Table shows the comparison between original word-list and the respective WordNet lexicon based on count frequencies, excluding sentences where neither positive nor negative words appear. Mean is the arithmetic mean, SD is the standard deviation, Corr is the Pearson correlation coefficient, N is the number of observations.

After a positive evaluation of WordNet lexicons, we move to the next step and evaluate the equivalence of French lexicons with their English originals. We build French lexicons from the same synsets as the English lexicons, following the steps described in the methodology section. Later, we correct them manually by removing erroneous and unwanted lemmas, while adding select missing lemmas. The French versions of LM lexicons include 145 and 260 lemmas in the positive and negative list, respectively, after removing lemmas that occur fewer than ten times. In the case of Henry, the French lexicons count 82 and 57. Thus, we note again a reduction of lexicon length compared to their originals, which is particularly visible in the LM case, mainly because many lemmas included in the original list do not appear in the corpus with sufficient frequency.

Table 4 presents relative frequencies of thirty top lemmas in each French lexicon. A comparison with Table 1, which contains the results from English lexicons, reveals that the same concepts are included in both lists, but the frequencies differ. The lemma “strong”, for example, appears with a relative frequency of 31 in the English corpus, but the French equivalent “fort” appears with a frequency of just 21. In a similar vein, “croissance” is more frequent than “augmentation” in French, whereas in English the order is reversed: “growth” is less common than “increase” In addition, another French lemma “solide” from the same synset, “solide.s.07”, appears with a frequency of 20, whereas in the English corpus “solid” appears with a low frequency. These differences are examples of cross-language variations in the preference given to particular words, which underscores the need to consider multiple synonyms rather than selecting the closest equivalent. Overall, the concentration of frequencies in each list appears similar to the English versions. Statistical properties are thus carried forward from English lexicons to the French ones.

**Table 4:** The top 30 lemmas in the French lexicons.

Loughran-McDonald				Henry et al. [10]			
**Positive**		**Negative**		**Positive**		**Negative**	
hausse	100	risque	100	important	100	risque	100
bon	77	obligation	77	croissance	77	baisse	44
permettre	70	perte	76	recevoir	71	diminution	35
élevé	68	clôture	39	bénéfice	69	réduire	33
grand	59	diminution	35	augmentation	63	réduction	25
haut	50	réduire	33	réaliser	54	diminuer	24
atteindre	43	réduction	25	présenter	54	passer	19
supérieur	42	diminuer	24	augmenter	53	défavorable	17
avantage	35	forme	19	bon	52	faible	17
bien	33	priver	18	élevé	46	négatif	14
accroître	33	défavorable	17	grand	39	petit	10
assurer	32	faible	17	haut	33	distribuer	9
occasion	31	écart	17	développement	30	incertitude	9
premier	31	entraîner	16	atteindre	29	effort	7
amélioration	26	recherche	15	supérieur	28	difficile	5
améliorer	22	restriction	15	dirigeant	28	baisser	5
favorable	22	différer	15	avantage	23	problème	5
spécial	22	négatif	14	bien	22	faiblesse	5
fort	21	restructuration	14	occasion	21	amortir	4
exclusif	21	type	11	élever	18	abandonner	4
meilleur	21	délai	10	amélioration	18	faillite	3
solide	21	renvoi	10	partir	16	difficulté	3
positif	20	incertitude	9	améliorer	15	récession	3
respecter	19	régler	9	fort	14	chute	3
remplacer	17	limite	9	meilleur	14	éviter	2
modèle	16	limiter	8	solide	14	chuter	2
effectif	14	défaut	8	positif	14	refuser	2
suivre	14	retenir	8	remettre	12	modeste	2
dépasser	13	fermeture	8	suivre	9	défi	2
faveur	13	remise	7	dépasser	9	risquer	2

*Note:* Table shows relative counts, setting the top most frequent lemma to count 100.

We proceed to evaluate equivalence based on the comparison of classification results of parallel English and French sentence pairs in Table 5. The results are understandably weaker than for English lexicons. Still, weighted macro F1-scores beat the 0.70 mark, the lowest being 0.74 for the positive Henry's lexicon, and the highest 0.92 for the negative Henry's lexicon. Precision, ranging from 0.54 to 0.80, is at similar levels as recall, ranging from 0.54 to 0.63. The proportion of sentences containing positive lemmas is slightly lower than those with negative lemmas in LM lexicons, where the negative list is much longer. In the case of Henry, where the two lists are of comparable length, about twice as many sentences are classified as positive, compared to negative ones. This is in line with classification results for sentences in English

**Table 5:** Classification equivalence between English and French lexicons.

	Precision	Recall	F1	Macro F1	Kappa
Loughran-McDonald					
Positive	.54	.63	.58	.75	.40
Negative	.69	.54	.60	.77	.45
Net-Positive	.70	.84	.77	.76	.53
Net-Negative	.84	.71	.77	.76	.53
Henry et al. [10]					
Positive	.65	.56	.61	.74	.42
Negative	.80	.60	.69	.92	.64
Net-Positive	.87	.91	.89	.85	.65
Net-Negative	.79	.72	.76	.85	.65

*Note:* Table shows the comparison between classification results between a WordNet lexicon in English (true values) and its French equivalent (predicted values) based on a parallel corpus of 69,913 sentences. Precision is the ratio of true positives to all positives, Recall is the ratio of true positives to the sum of true positives and false negatives, F1 is the harmonic mean of precision and recall, Macro F1 is the average of scores for all classes (e.g. positive and non-positive), Kappa is Cohen's kappa score.

When we calculate net-positivity ratios and use them to classify sentences as either positive or negative, depending on the sign, we achieve macro weighted F1-score values of 0.76 for LM, and 0.85 for Henry. Precision and recall ratio values are better balanced than for simple classification based on the presence of either a positive or negative lemma described above. Cohen's Kappa values are 0.53 and 0.65, respectively, again higher than reported in the paragraph above. The proportion of net-positive to net-negative sentences is below 1:1 for LM but above 2:1 for Henry lexicons. These values resemble the results we obtained for English lexicons.

Finally, we examine the value of net-positivity ratio values, as presented in Table 6. The mean values are somewhat different but the standard deviations are relatively close, indicating a good fit between the two language versions. We observe a slight bias towards the positive in the French corpus compared to the parallel English sentences (by a fraction of the standard deviation). Still, the sample being large, the difference is statistically significant. Overall, ratio values correlate between the two language versions, obtaining the Pearson coefficient of 0.62 for LM, and 0.75 for Henry. Given a large sample size, the correlation is thus positive and significant. In sum, the results attest to a positive evaluation of equivalence between measures obtained from English and French texts.

**Table 6:** Comparison of Net-Positivity scores between English and French lexicons.

	Mean	SD	Corr.	N
Loughran-McDonald				
English lexicon	−0.08	.88	.62	23,707
French lexicon	.10	.88		
Henry et al. [10]				
English lexicon	.38	.81	.75	18,382
French lexicon	.43	.81		

*Note:* Table shows the comparison between original word-list and the respective WordNet lexicon based on count frequencies, excluding sentences where neither positive nor negative words appear. Mean is the arithmetic mean, SD is the standard deviation, Corr is the Pearson correlation coefficient, N is the number of observations.

## Conclusions

This paper presents a method for translating financial lexicons from English, where they are well established, to French, which we selected as an example, using available python packages and code that we publish on https://github.com/kmk4842/opus2021. WordNet makes it possible to automize this task which used to be conducted manually [17] or using machine translation González et al. [16]. The new method allows us to represent the lexicons as sets of word senses rather than sets of word forms. We then use the meaning implied in the relationship between words belonging to one synset in WordNet to obtain relevant words in French via interlingual mapping. Validation results are satisfactory, showing a promising degree of equivalence between sentiment classifications and net-positivity ratios in pairs of parallel English and French sentences. The same steps can be followed to obtain comparable measures across other language pairs.

Researchers may use our method in studies involving a multilingual setting, and cross-linguistic studies in the international setting. In particular, we hope to encourage studies in developing countries and promote sustainable development initiatives beyond the English-speaking world. Using the python code that we provide, researchers can readily obtain comparable measures of sentiment for various languages included in OMW. Nevertheless, the method has some limitations: it relies on the vast but still limited knowledge base of OMW, manual verification and correction remains necessary, and a parallel corpus is needed for validation. Further research may lead to mapping tasks for additional languages, and it may improve upon our method.

## Related research article

N/A

## Ethics statements

No ethical issues were identified in this study because it relies on data made publicly by companies as required by law in Canada.

## CRediT authorship contribution statement

**Karol Marek Klimczak:** Supervision, Funding acquisition, Conceptualization, Methodology, Validation. **Jan Makary Fryczak:** Software. **Dominika Hadro:** Methodology, Writing – original draft. **Justyna Fijałkowska:** Resources, Writing – review & editing.

## Declaration of competing interest

The authors declare that they have no known competing financial interests or personal relationships that could have appeared to influence the work reported in this paper.

## Data Availability

The authors do not have permission to share data.
